# What is more likely in orthorexia nervosa: perfectionism or OC symptoms? A bayesian method in clinical and non-clinical samples

**DOI:** 10.1186/s40359-025-02517-2

**Published:** 2025-03-11

**Authors:** Caterina Novara, Eleonora Maggio, Massimiliano Pastore, Sara Piasentin, Susanna Pardini, Sofia Mattioli

**Affiliations:** 1 Department of General Psychology, University of Padova, Via Venezia 8, Padova, 35131 Italy; 2 Department of Developmental Psychology and Socialization, University of Padova, via Venezia 8, Padova, 35131 Italy; 3 Fondazione Bruno Kessler, Digital Health Research, Centre for Digital Health and Wellbeing, Via Sommarive 18, Trento, 38123 Italy

**Keywords:** Orthorexia nervosa, Perfectionism, Obsessive-compulsive symptoms, Risk factors

## Abstract

**Purpose:**

Orthorexia Nervosa (ON) exhibits specific features that may overlap with Obsessive-Compulsive Disorder (OCD), Perfectionism Striving (PS), and Perfectionism Concern (PC). While previous literature has shown predictiveness in different characteristics of ON, this study aimed to determine if PS, PC and OCD symptoms could predict ON dimensions in at-risk populations using Bayesian models.

**Method:**

The study enrolled 622 individuals from three different at-risk populations: people who were following treatment for an Eating Disorder (Patients), people who were following a diet (Dieters) and University students with a degree in medicine or nursing (Students).

**Results:**

Using Bayesian probabilistic methods and considering group factors, the model was enhanced highlighting that ON characterised Patients, Dieters, and Students. The predictiveness of OC features, PS, and PC in different ON characteristics was confirmed in each group, and different patterns were observed in the three groups. Regarding problems related to ON, predictors were higher in Patients, followed by Dieters and Students. Posterior Predictive Distribution (PPD) showed that almost 50% of Patients incur ON-related problems. In ON knowledge, Patients and Dieters are very similar. When feelings related to ON were considered, Patients and Dieters showed different interactions.

**Conclusion:**

Clinicians should consider that one in two patients suffering from EDs might develop ON-related problems. People on a diet could show similar patterns of symptoms to patients in ON knowledge and feelings. Finally, our results confirm that perfectionism represents a risk factor for ON in each group considered.

**Supplementary Information:**

The online version contains supplementary material available at 10.1186/s40359-025-02517-2.

## Introduction

Orthorexia Nervosa (ON), first described by Steven Bratman [1], is a disordered eating condition characterized by an obsessive focus on healthy eating [2]. Individuals with ON exhibit an excessive preoccupation with food quality, adhering to rigid and inflexible dietary rules. These self-imposed rules often demand significant time and effort to research, plan, acquire, prepare, and consume meals. Furthermore, individuals with ON may entirely exclude certain food groups they perceive as “unhealthy,” potentially leading to nutritional deficiencies. ON is also associated with emotional distress, such as guilt after consuming foods deemed unhealthy, cognitive impairments like difficulties in concentration due to intrusive food-related thoughts, and social withdrawal from events involving food [3]. Despite increasing academic interest in ON, it is not yet recognized in diagnostic manuals for mental disorders [3].

The lack of consensus on ON diagnostic regards some shared characteristics between ON and other psychopathological conditions; furthermore, difficulties in making agreements on ON as a diagnostic category raises debates about whether ON should be classified as an Eating Disorder (ED), Obsessive-Compulsive Disorder (OCD), or a distinct condition related to maladaptive eating habits [4].

ON shares key features with EDs, including perfectionism, concern for being overweight, and orientation to appearance [5]. Similarly, ON and OCD both involve intrusive thoughts about food and health, concerns about contamination, and ritualized food preparation and consumption, alongside perfectionistic tendencies [2, 6, 7]. Therefore, the most frequent obsessions regard contaminations or harmful thoughts, whereas checking and washing rituals represents the most frequent compulsions [8, 9] However, literature has highlighted stronger parallels between ON and EDs than between ON and OCD [10–12] A recent meta-analysis highlighted that ON symptoms are more frequently associated with EDs than OCD; moreover, this study put in evidence that ON is distinct from EDs or OCD disorders [13]. A systematic review further suggests that ON may represent a separate category of ED [14].

EDs are considered a risk factors for ON, with previous diagnoses of conditions like Anorexia Nervosa (AN) [15], body dissatisfaction, or a dysfunctional idea of thinness are linked with greater ON problematic behaviors, while OC characteristics were not related [16]. Moreover, as the literature shows diet is a risk factor in the development of EDs [17], interest in the relationship between diet and ON has also increased. It has been observed that individuals following a vegetarian diet have greater orthorexic behaviors or a greater risk of developing ON [18], and ON seems to be associated with the number and type of diets followed over a lifetime [16]. Perfectionism plays a critical role here, as individuals who voluntarily follow diets and exhibit perfectionistic traits are more prone to ON [10]. Certain groups, such as medical students [19] and nutrition/dietetics students [20], are particularly at risk. Perfectionism represents an important factor related to ON, EDs, OCD, and diet. Perfectionism is a personality and multidimensional construct [21–23] related to high personal performance standards, strivings for flawlessness, and the tendency to be overly critical of one’s behavior. Reviews identify two higher-order dimensions of perfectionism: Perfectionistic Strivings (PS) and Perfectionistic Concerns (PC) [24]. PS is mostly self-oriented and regards the attempt to pursue perfection as consistent with high personal standards. PS shows relationships both with “maladaptive” (passive coping styles) and “adaptive” (active coping styles) outcomes [25]. PC, in contrast, is socially driven, characterized by fear of failure, concerns over mistakes, and a perceived gap between achievements and expectations. PC is considered maladaptive and linked to negative outcomes. Considering these facets, perfectionism could play a central role in a broad range of symptoms and a wide variety of psychopathologies [21]. In OCD, perfectionism is recognized as a risk factor [26], and its association with OCD has been well-documented [27]. In EDs, perfectionism is both a precursor and a maintenance factor [28]: it is described as a personality characteristic that precedes EDs, and that could lead to a worse prognosis [29, 30]. Two meta-analyses on adults [31] and on children/adolescents [32] have confirmed that total perfectionism, PS, and PC had significant positive associations with EDs. In addition, perfectionism has been highlighted as a non-adaptive characteristic that is likely to exacerbate OCD and ED symptoms [33]. Therefore, perfectionism is a transdiagnostic construct displayed both by ON, EDs, and OCD [7].

ON shows relationships with different facets of perfectionism (as a personality trait related to EDs and OC features) [34]. Studies have shown that self-oriented, others-oriented, and socially prescribed perfectionism correlate with ON [35]. Moreover, perfectionism was positively correlated with all aspects of ON facets [36]. The same results highlighted that perfectionism predicted ON in the groups of people on a diet, and it was statistically significantly higher in the group with high ON compared to the group also on a diet but with low ON [12].Moreover, studies supported the previous findings and highlighted that perfectionism was positively and indirectly associated with ON characteristics via biased beliefs about maladaptive healthy eating strategies [37].

While perfectionism is a defining characteristic of EDs and OCD [38], its role in ON requires further exploration, especially concerning perfectionism concerns, perfectionism strivings, and their relation with OC symptoms. Because the two dimensions of perfectionism have shown different relationships (i.e [39]), the differentiation between PS and PC is essential for understanding the correlates and consequences of perfectionism. Based on existing literature, perfectionisms and OCD symptoms are often associated with EDs, however the specific relationship between those constructs in ON remains unclear. This study aims to develop a Bayesian probabilistic model to explore the role of PC, PS, and obsessive-compulsive symptomatology in ON across three at-risk groups: individuals with EDs, those on diets, and university students. We aim to understand if perfectionism and OC symptomatology are determining variables to explain the model in different at-risk groups.

Our main hypothesis are as follows:

- Higher perfectionistic concerns are expected to be associated with increased ON behaviors.
- Perfectionistic striving is predicted to influence feelings and knowledge related to ON.
- It is anticipated that the clinical group will score higher on ON measures compared to the diet and university student groups.
- Clinical and diet groups are expected to exhibit more dysfunctional beliefs about healthy eating and maladaptive feelings about maintaining a healthy diet compared to university students.
- The clinical group is predicted to show higher levels of perfectionistic concerns than the other two groups.
- No significant differences in perfectionistic striving are expected among the groups.

## Methods

### Participants

The total sample consisted of 622 adults selected from three different at-risk groups (university students, patients suffering from EDs, people on diet), that voluntarily participate in the study. Individuals belonging to three groups named “university students’ group”, “clinical group”, and “diet group”. Data of this sample have been already utilised in a larger research [10, 12, 40].

The student group is composed of 399 (64.1%) students in medicine, physiotherapy, and nutrition who were recruited in northern Italy, using the snow-ball sampling. The initial students group recruited provided the contacts of other university students, subsequently contacted to take part in the present research. The group was characterized by 272 (68.2%) females with a mean age of 21.58 (SD = 3.70, ranging from 18 to 49 years), and a mean of 15.38 years of education (SD = 1.84, ranging from 8 to 25). This group had a BMI mean of 21.23 (SD = 2.72, ranging from 16 to 38). Three hundred and seventy-eight participants (95%) were single or in a relationship but did not live together.

The clinical group was composed of 140 (22.5%) patients hospitalised in an Eating Disorder Treatment Centre in central and northern Italy; they completed the instruments of assessment during their hospitalisation. All people of this group had received a diagnosis of DSM-5 Eating Disorders (EDs) by the clinicians: 54 (38%) with Anorexia Nervosa, 11 (7.7%) Anorexia Nervosa Binge-Eating/Purging subtype (ANBP), 16 (11.3%) with Binge Eating Disorder (BED), 20 (14.2%) with Bulimia Nervosa, 4 (2.8%) with Other Specified Feeding and Eating Disorders (OSFED; and 35 (25%) Eating Disorder not Otherwise Specified (EDNOS). Clinicians formulated diagnosis based on the DSM-5 and used standardized assessment measures. 123 (87.9%) were female, with a mean age of 37.24 (SD = 17.66; ranging from 18 to 74 years), a mean of 14.09 years of education (SD = 3.46, ranging from 5 to 27), and a BMI mean of 25.76 (SD = 11.47, ranging from 11 to 61). Eighty-seven participants (62.1%) were single or in a relationship but did not live together. Thirty-nine (27.9%) were students.

The diet group consists of 83 (13.3%) individuals who were following a diet at the time they had filled out the present research questionnaires; they were following a “zone diet” (a diet which claimed to reduce body inflammation, based on reduced carbohydrate consumption) prescribed by a northern Italy dietician. Participants at home completed the questionnaires, which had to be delivered to the doctor. This group was characterized by 46 (55.4%) female, with a mean age of 45.10 (SD = 12.93; ranging from 18 to 68 years), a mean of 14.09 years of education (SD = 2.95, ranging from 8 to 20), and a BMI mean of 24.86 (SD = 5.71, ranging from 16 to 45 years). Twenty participants (24.1%) were single or in a relationship but did not live together. Forty-three (51.8%) were occupied with a full-time job.

Individuals participated voluntarily and gave their written consent before taking part in the study. Participants were fully informed about the research; moreover, the anonymity and confidentiality of the collected data were guaranteed. They responded to a series of paper-and-pencil self-report questionnaires in a session that lasted approximately 20 min. All measures were administered in a counterbalanced order to avoid any order effects. The study adhered to the principles outlined in the Declaration of Helsinki and received approval from the Ethical Committee of the Department of General Psychology at the University of Padova (EC436767EoECD1BCBE97F59A88EB2D59).

### Measures

The **Eating Habits Questionnaire** (EHQ-21) [41], Italian version by [42] is a 21-item self-report questionnaire designed to assess various characteristics of ON using a four-point Likert scale. In the EHQ-21, higher scores correspond to higher presence of ON features. It is divided into three subscales named “Knowledge”, “Feelings” and “Problems”. The “Knowledge” subscale assesses the belief of one’s superiority in the knowledge of healthy eating and in pursuing a better diet than others. Also, it evaluates the belief of one’s superiority in preparing meals in the healthiest way possible. The “Feelings” subscale assesses the personal satisfaction derived from adherence to healthy eating habits, as well as the sense of having control over one’s diet and the efforts made to attain it. The “Problems” subscale evaluates the consequences of orthorexic behaviors on social and work functioning, the presence of obsessions and excessive worries about healthy eating, and the problems derived from following strictly a certain type of diet. The original and the Italian versions of the EHQ-21 demonstrate good psychometric properties. Cronbach’s alpha for each subgroup was calculated (0.70 < Cronbach’s alpha < 0.92).

The **Multidimensional Perfectionism Scale** (MPS) [21, 43], Italian version by [44] is a self-report questionnaire composed of 35 items that assess perfectionism on a five-point Likert scale. Higher scores on MPS correspond to higher levels of perfectionism. Authors identified two different dimensions of perfectionism: the dimension of positive strivings expressed by the “Personal Standards” scale and a negative perfectionism consisting of maladaptive concerns about actions expressed by the “Concern over Mistakes” and “Doubting of Actions” subscales. Cronbach’s alpha for subscales was calculated (0.56 < Cronbach’s alpha < 0.63).

The **Obsessive Compulsive Inventory-Revised** (OCI-R) [45]; Italian version by [46] is a self-report questionnaire that measures symptoms of Obsessive-Compulsive Disorder. Higher scores on the OCI-R correspond to more severe OCD symptoms. The questionnaire comprises 18 items divided into six subscales: “Washing”, “Ordering”, “Hoarding”, “Mental Neutralizing”, “Obsessing” and “Checking”. Cronbach’s alpha for each subgroup was calculated (0.75 < Cronbach’s alpha < 0.92).

### Statistical analysis

Bayesian statistics were utilized for all the analyses as they allow to incorporate all the unknown parameters that can be defined by a probability distribution. Bayesian statistics differ from classic statistics as information from prior research can be included in the model without testing the same null hypothesis [47].

The analyses were performed using the statistical software R [48], along with the brms package [49, 50], which integrates STAN for implementing Markov chains Monte Carlo (MCMC) sampling [51], and ggplot2 [52] packages. Initially, univariate and bivariate distributions of the target variables were explored. Subsequently, a series of multivariate linear models were analyzed using a Bayesian approach to estimate parameters.

### Priors specification

The number of parameters in these models ranges from a minimum of 9 – i.e., intercepts, standard deviations and residual correlations in the null model (M00) – to 42 in the model with GROUP interactions (M05).

Prior probability distributions were defined for each model parameter to formalize prior hypotheses. In particular, we adopted the Student’s t for intercepts, regression coefficients, and residual standard deviations and the *Lewandowski-Kurowicka-Joe* (LKJ) [53] for residual correlations (Table 1 in Supplementary Material). Models Analysis Plan.

For the analyses we proceeded with the following steps:

1. **Fitting models.** We fitted all models using the specified priors (Table 2 in Supplementary Material). For MCMC sampling we adopted 4 chains of 2000 replicates each, with 1000 as warmup. Consequently, the effective number of posterior samples was 4000. MCMC convergences were assessed by means of the Potential Scale Reduction Statistic (PSRF or Rhat) [54].
2. **Diagnostics.** In addition to the traditional residuals diagnostics plots (for linearity, normality and homoscedasticity), we adopted the *Posterior Predictive Check* (PPC), this latter is a graphical comparison between data simulated from the posterior predictive distribution and real-world observations; if a model is a good fit then generated data looks a lot like the observed data [55].
3. **Model comparison.** We used the following indices: *Leave-one-out cross validation criterion* (LOO) [56], *model weights* (W) [57] and R^2^ – one for each response variable – with 89% *credible intervals.* Model weights are values ranging from zero to one that can be interpret as *an estimate of the probability that the model will make the best predictions on new data*,* conditional on the set of models considered* [58]. Therefore, in the set of considered models, the best will be the one with lowest LOO and highest W.
4. **Best Model analysis.** Finally, we selected the best model and analyzed it in detail. In particular, we analyzed the model predictions based on the parameters posterior distributions and the *Posterior Predictive Distribution* (i.e. the distribution of possible unobserved values conditional on observed data and model parameters, PPD; [59]).

## Results

In Table 1 are reported the univariate statistics for the quantitative target variables. For GROUP, that is a categorical variable, we had 399 subjects in the Students group, 140 subjects in the Patients group and 83 subjects in the Dieters group.

**Table 1:**
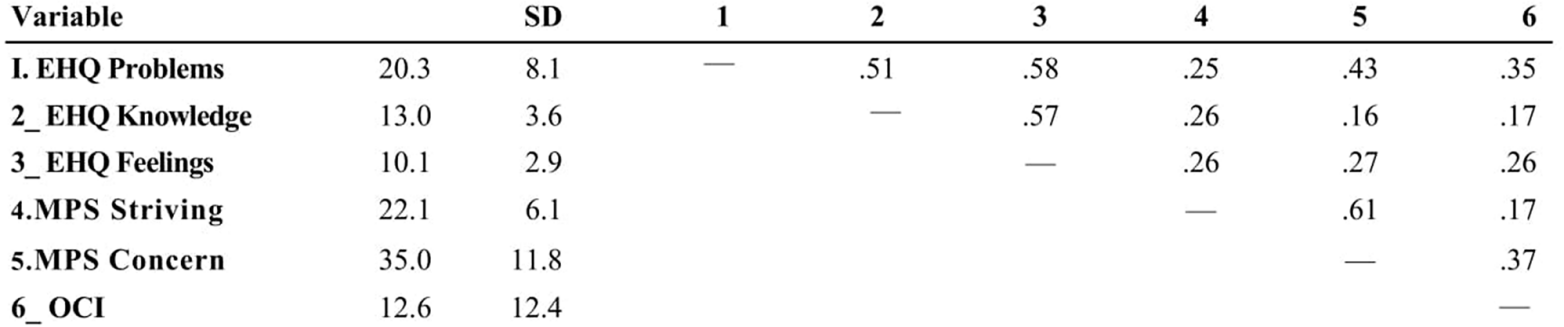
Descriptive statistics and inter-correlations between quantitative variables (*N*=622)

### Model comparison

Table 2 summarizes the model comparison. The best model was M05 – Y ∼ GROUP × MPS striving + GROUP×MPS concern + GROUP×OCI – with LOO = 9818.8 (s.e. 69.3), W = 0.86 and a relative evidence of about 8.83 times that of the second ranked model (M03). This latter was obtained dividing the two models weights, 0.86/0.1. In other words, including interactions with GROUP improved over eight times the simple additive model (M03). Model M05 presented also the higher values of R2 in all endogenous variables, 0.49 for EHQ Problems, 0.25 for EHQ Knowledge and 0.25 for EHQ Feelings, respectively.

**Table 2:**
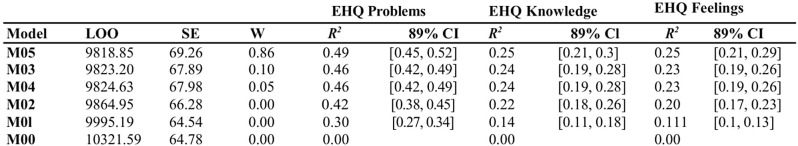
Model comparison table. LOO=Leave-one-out cross validation criteria; SE= standard error; W= model weight; R2=R^2^, CI= 89% credible interval

### Best model analysis

From this point, we focused solely on the best-fitting model (M05). After performing the diagnostics on residuals and the PPC, we analysed the model predictions and estimated the posterior probabilities related to our hypotheses. For clarity, the results are presented separately for the three endogenous variables. Figures 2 and 4, and 6, represent the conditional expected values, with relative 89% Credible Intervals, of the three model interactions on the three endogenous variables: EHQ Problems, EHQ Knowledge, and EHQ Feelings, respectively. X-axes represent the predictors – MPS striving, MPS concern, OCI TOT–, colours refer to the groups. Figures 3 and 5 represent the marginal Posterior Predictive Distributions (PPD; [59]) of groups scores or difference between group scores; using these PPD we computed the posterior probabilities related to our hypotheses.

We hypothesized that the Patients group would have higher scores because it is composed of patients with a diagnosis of EDs. Moreover, we aim to understand if the model fits with other groups, adding dieters and university students; our hypothesis is that there is a decreasing gravity of symptoms in the three groups (Patients > Dieters > Students). Defining priors, we expected for EHQ Problems (the subscale ranging from 0 to 48 points) that a clinical subject has a score greater than 32 and a difference of at least 4 points with other groups. For EHQ Knowledge (the subscale ranging from 0 to 20 points) a clinical subject has a score greater than 10 and has a difference of at least 2 points from other groups. Finally, for EHQ Feelings (the subscale ranging from 0 to 16 points) that a clinical subject has a score greater than 8 and has a difference of at least 2 points with other groups. Those scores have been chosen since no cut-off has been established.

**Fig. 1:**
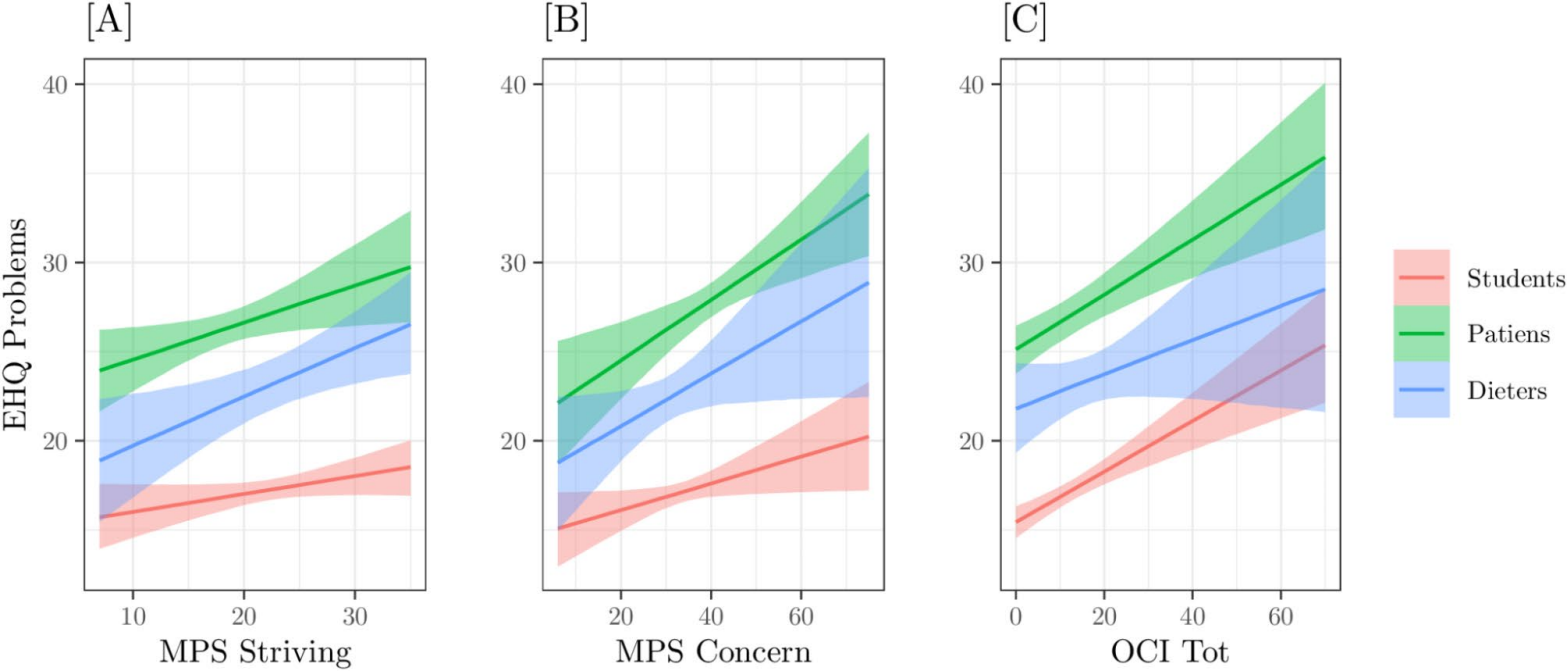
Model M05 predictions on variable EHQ_PROBLEMS as a function of [**A**] group x MPS positive, [**B**] group x MPS Negative, [**C**] group X OCI. Bands indicate the 89% Credible Interval of expected values

**EHQ Problems.** On this dependent variable, the interactions involving the group variable were not particularly pronounced, as the slopes for all three predictors were nearly parallel across groups Fig. 1). The order of expected scores with respect to groups also remains the same with clinicians having higher values, followed by the Dieters group and then the Students group. For MPS striving Fig. 1[A]), the Students group has a slope (β = 0.03) slightly lower than that of the other two groups − 0.06 in the clinical and 0.08 in the diet respectively; for MPS Concern Fig. 1[B]) the slopes are 0.05 (Students), 0.12 (Patients) and 0.1 (Dieters), respectively, supporting the initial hypothesis that negative perfectionism has a greater impact than positive perfectionism; for OCI TOT Fig. 1[C]) the slopes are 0.1 (Students), 0.1 (Patients) and 0.07 (Dieters), respectively, sustaining the assumption that OC symptomatology is a determining variable for problems related to ON both in Students group, in Patients group, and in Dieters group.

**Fig. 2:**
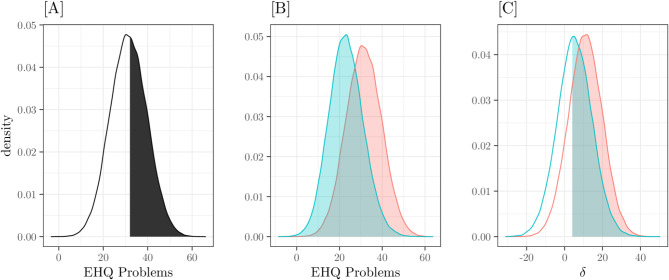
EHQ_PROBLEMS. Posterior Predictive Distributions. [**A**] Patients group scores; the black area is *Pr*(x > 32) = 0.47. [**B**] Scores of the Patients group (in red) and the other two groups; *Pr*(x_Patients_ > x_others_) = 0.23. [**C**] Differences between Patients-Students scores (red line) and between Patients-Dieters scores (light blue line): the filled areas are *Pr*(δ_Patients−Students_ > 4) = 0.78 and *Pr*(δ_Patients−Dieters_ > 4) = 0.56

Figure 2 depicts the PPD of EHQ PROBLEMS scores. Panel [A] shows the density of clinical subjects’ posterior scores; using this distribution, we can estimate the posterior probability that a clinical subject’s score is greater than 32 – the black area – which is 0.47. Panel [B] shows the density of the Patients group posterior scores (red density) compared with the density of the pooled scores of the other two groups; from these two distributions, we can compute the Probability of Superiority – i.e., the posterior probability that the score of a clinical subject be greater than the scores of subjects belonging other groups – that is 0.23.

Panel [C] represents the posterior distributions of score differences Patients-Students (red) and Patients-Dieters (lightblue); colored areas represent the probability that differences in scores between Patients-Students and Patients-Dieters be greater than 4 is 0.78 and 0.56, respectively.

**Fig. 3:**
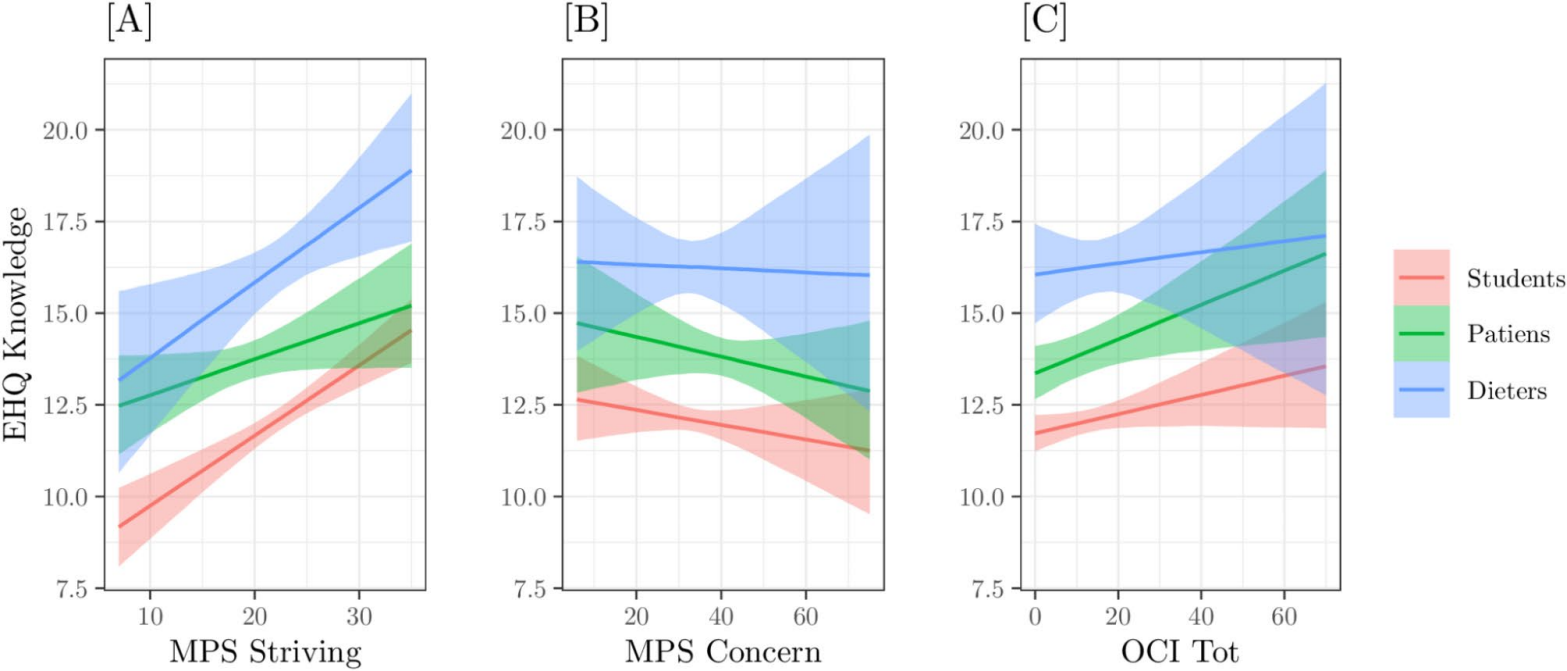
Model M05 predictions on variable EHQ_KNOWLEDGE as a function of: [**A**] group x MPS positive, [**B**] group x MPS negative, [**C**] group x OCI. Bands indicate the 89% Credible Interval of expected values

**EHQ Knowledge.** In this dependent variable, we observe different interactions between predictors and groups. In general, the relationship between MPS striving Fig. 3[A]) and OCI TOT Fig. 3[C]) is positive, with MPS concern Fig. 3[B]) is negative; the straight line with higher expected values is always those of Dieters group, followed from those of Patients group and finally those of Students group, which has the lowers expected values. Slopes concerning the MPS striving were: 0.05 for the Students group, 0.03 for the Patients group, and 0.06 for the Dieters group Fig. 3[A]); with respect to MPS concern were − 0.02 for the Students group, -0.02 for the Patients group, and − 0.01 for the Dieters group Fig. 3[B]); concerning the OCI TOT slopes were: 0.02 for the Students group, 0.03 for the Patients group and 0.01 for the Dieters group Fig. 3[C]).

**Fig. 4:**
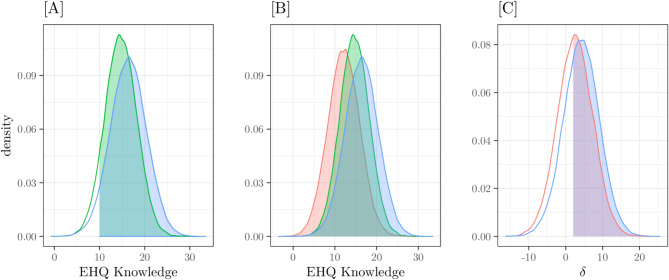
EHQ_KNOWLEDGE. Posterior Predictive Distributions. [**A**] Clinical group scores - *Pr*(x > 10) = 0.9 (green area) - and Dieters group scores - *Pr*(x > 10) = 0.95 (light blue area). [**B**] Scores of the three groups: clinical (green), Dieters (light blue) and population (red); *Pr*(x_Patients/Dieters_ > x_Students_) = 0.72. [**C**] Differences between Patients-Students scores (red line) and between Dieters-Students scores (light blue line); the filled areas are *Pr*(δ_Patients−Students_ > 2) = 0.54 and *Pr*(δ_Dieters−Students_ > 2) = 0.66

The marginal PPD is related to the target hypotheses (Fig. 4). Panel [A] represents scores of Patients (green) and Dieters (light blue) groups. Colored areas indicate the estimated probability that scores greater than 10 are 0.9 for patients and 0.95 for dieters, respectively. Panel [B] also includes the scores of the Students group (red); the probability that a subject belonging to either group Patients or group Dieters has scores greater than those of a subject of group Students was 0.72. Finally, panel [C] represents the PPD of score differences between Patients-Students (in red) and Dieters-Students (in light blue); colored areas represent the probability that such difference be greater than 2 is 0.54 and 0.66, respectively.

**Fig. 5:**
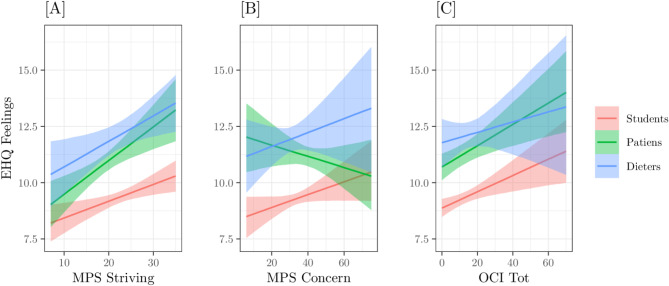
Model M05 predictions on variable EHQ_FEELINGS as a function of: [**A**] group x MPS positive, [**B**] group x MPS negative, [**C**] group x OCI. Bands indicate the 89% Credible Interval of expected values

**EHQ Feelings.** For this dependent variable the interaction effects appeared stronger Fig. 5). The Student group presents the lowest expected values with a positive slope in all three predictors, with slopes of about 0.02.In the other two groups, we observed different patterns. In the MPS striving predictor Fig. 5[A]), the Dieters scores are always higher, but the difference with the Patients group decreases as the predictor values increase; slopes were 0.03 for Dieters and 0.04 for Patients, respectively. About the MPS concern Fig. 5[B]) the Dieters group has lower scores than the Patients group for low values of the predictor as the relationship reverses as it increases; for Dieters the relationship is positive (0.02) while for Patients it is negative (-0.02). In relation to the predictor OCI TOT Fig. 5[C]) we observe an inverse situation to that of MPS concern i.e., the Dieters group scores are first higher and then lower than those of the Patients group; slopes were 0.02 and 0.03, respectively.

**Fig. 6:**
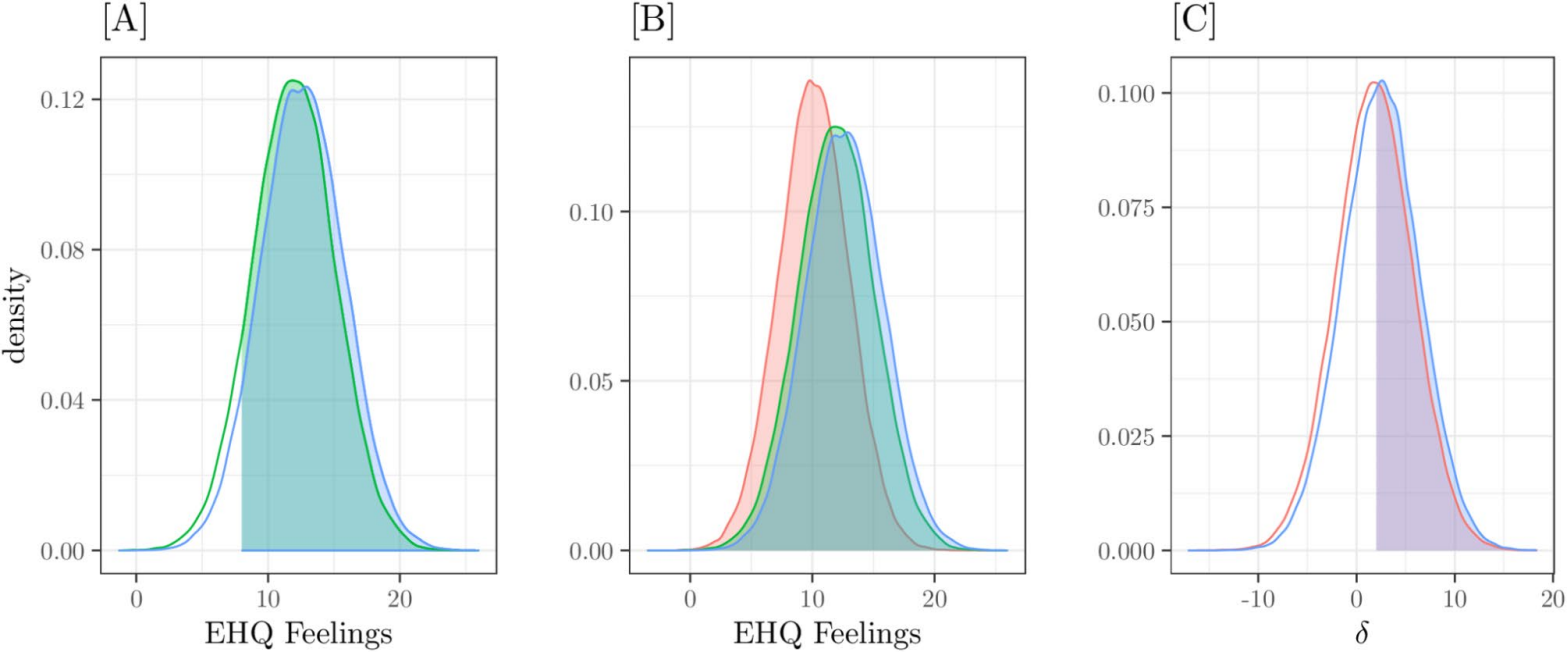
EHQ_FEELINGS. Posterior Predictive Distributions. [**A**] Clinical group scores - *Pr*(x > 8) = 0.9 (green area) - and Dieters group scores - *Pr*(x > 8) = 0.93 (light blue area). [**B**] Scores of the three groups: clinical (green), Dieters (light blue) and population (red); *Pr*(x_Patients/Dieters_ > x_Students_) = 0.69. [**C**] Differences between Patients-Students scores (red line) and between Dieters-Students scores (light blue line); the filled areas are *Pr*(δ_Patients−Students_ > 2) = 0.49 and *Pr*(δ_Dieters−Students_ > 2) = 0.53

Finally, marginal PPDs related to the hypotheses of interest are depicted in Fig. 6. In panel [A] are the EHQ FEELINGS scores of the Patients group (in green) and the Dieters group (in light blue). The filled areas indicate the estimated probabilities that the scores are greater than 8, respectively 0.9 for Patients and 0.93 for Dieters. In panel [B], the density of PPD relative to the Students group (in red) was also added; the probability of a subject in the Patients/Dieters groups having a higher score than a Students subject (Probability of Superiority) is 0.69. Panel [C] shows the PPDs of the differences in the Patients-Students (in red) and Dieters-Students (in light blue) scores; the filled areas represent the probabilities that this difference is greater than 2, and are respectively 0.49 and 0.53.

## Discussion

The primary aim of the currentstudy was to explore the relationship between perfectionism and obsessive-compulsive symptomatology in individuals with Orthorexia Nervosa (ON). Specifically, it investigated how different aspects of perfectionism, such as concerns and striving, impact ON-related behaviors, beliefs, and feelings. By comparing three groups at risk (clinical, diet, and university student groups), the study sought to identify the best-fitting model to explain these relationships. The findings emphasize the complex interplay between perfectionism, OC symptoms and ON characteristics (Problems, Knowledge, Feelings), with distinct patterns observed across the three groups. Key findings include:

- Perfectionistic Concerns (PC) had a stronger impact on ON than Perfectionistic Strivings (PS).
- OC symptoms were a significant predictor of problems related to ON across all groups.
- Clinical group exhibited the highest levels of ON, dieters showed high levels of ON especially in terms of knowledge and feelings related to ON, while students had the lowest levels of ON, but their scores increased with higher levels of perfectionism and OC symptoms.

Our results highlighted that including the group factor enhances our model over eightfold, confirming the hypothesis that ON is a characteristic of the clinical group [10–12, 15], the Dieters group and the Students group. Furthermore, perfectionism and OC symptomatology emerged as predictive variables for ON across all groups. This aligns with recent research showing that both perfectionism and obsessiveness, considered jointly, predict higher orthorexic tendencies in the general population [60]. Statistical analyses exploring correlations between the perfectionism dimensions and ON across distinct groups reinforced the necessity of including the group factor.

Considering EHQ-Problems, all dimensions of perfectionism and OC characteristics were significant predictors of ON-related problems. Referring to slopes, as expected, in all predictor variables, the higher values were shown by the Patients group, followed by the Dieters group, with the Students group displaying the lowest value. Specifically, considering the predictiveness of Perfectionistic Strivings (PS) for problems related to ON, it emerged that the three groups were independent of each other and that PS was more present in the clinical group and subsequently in the Dieters and the Students group. Perfectionistic Concerns (PC) also explained the problems related to ON in all groups, although it appeared to be more present in Patients group and significantly less in Students group. This result is coherent with literature [61, 62] as regards the role of maladaptive perfectionism in EDs and its multidimensional characteristic.

However, it was found that OC-symptomatology influenced the problems related to ON not only in the Patients group but also in others at risk, so it has an important impact on problems related to ON regardless of the group at risk. Thus, obsessive-compulsive characteristics represent an important factor in predicting ON problems but not in disentangling differences between different groups at risk. As a consequence, obsessive-compulsive aspects may be relevant in the construct of ON, but it could also be such a transversal construct ineffective in discriminating against groups at risk. The findings demonstrate that high levels of worry about mistakes, doubts about one’s actions, intolerance for imperfections, and the pursuit of perfection driven by high personal standards, contribute to negative consequences associated woth orthorexic behaviours. The effect of worries about performing tasks perfectly (PC) on problems was more pronounced in the Patients and the Dieters groups. At the same time, high personal standards (PS) influenced the three groups equally, even if the Students group showed lower levels of this perfectionism dimension.

The analysis of Posterior Predictive Distributions (PPDs) revealed that in 46% of cases, individuals in the Patients group exhibited high posterior scores for EHQ-Problems. These data underlined that a substantial proportion of individuals in the clinical group will report problems related to ON in the future. Furthermore, while **EHQ-Problems** partially differentiated between Students and Patients groups, it was ineffective in distinguishing between Patients and Dieters groups. This has an important clinical implication: Clinicians should closely monitor individuals who adhere to rigidly healthy diets, as a notable proportion may face ON-related issues or even be diagnosed with EDs. This finding underscores the dual need to clinically address individuals pursuing excessively strict healthy diets and recognize this behavior as a socially accepted yet potentially restrictive eating pattern. Future longitudinal studies in clinical ED samples should be conducted with this aim.

As regards EHQ-Knowledge, a dimension that expresses avoidance of “unhealthy” foods, concerns about their purity, beliefs on the superiority of one’s eating habits and intolerance towards other beliefs about eating, analyses highlighted different interactions between the three predictors and groups but always higher expected values (represented with a straight line) for the Dieters group, followed by the Patients and Students group. Considering the Striving dimension of perfectionism, we can detect overlaps between Dieters and Patients only for low values, for higher values solely Dieters have higher orthorexic knowledge. Interestingly, this result highlights that when scores on the positive component of perfectionism are lower, striving for perfection predicts orthorexic knowledge (characterised by excessive attention and rigid habits regarding eating healthy foods selected based on their quality) similar in Dieters and in Patients. However, for higher levels, PS predicts the presence of orthorexic knowledge only in the Dieters group. Dieters could be egosyntonic with their convictions about healthy diet so they do not worry and they gain their goals with the strict belief that they are following the proper eating habits; they are following voluntarily a diet and could believe that they have more information than others. This finding corroborates our prior research, which demonstrated that individuals who voluntarily adopt a diet exhibited significantly higher knowledge about orthorexia nervosa (ON) compared to those with a clinical diagnosis. This implies that dieters may perceive themselves as having a superior comprehension of healthy eating habits [10]. Additionally, Perfectionistic Striving could potentially be a cognitive factor contributing to the subsequent emergence of orthorexic symptomatology [40]. Perfectionism underlying orthorexic knowledge is a positive one related to high personal standards; that is confirmed by the result that the Students group reaches the clinical group in MPS-Striving. This results indicates that, not only among Dieters and in people with EDs, but also within the general population, high levels of PS are associated with orthorexic knowledge. Therefore, PS represents a risk factor for this construct related to orthorexia. Conversely, the Concern dimension of perfectionism (PC) exhibited an opposing pattern. Across all groups, PC demonstrated a negative relationship with orthorexic knowledge, failing to differentiate between groups at either low or high levels.

The three groups are different in EHQ-Knowledge for lower OC-symptoms, but there is an overlap between Dieters with both Patients and Students groups for higher obsessive-compulsive scores. Higher obsessiveness levels lead people to focus on overconcern about healthy eating habits; it could represent a cognitive strategy to ensure themselves and take control that they are pursuing the right way to eat healthy. The Posterior Predictive Distribution for EHQ-Knowledge revealed that while the slopes were low (indicating minimal variability), Dieters and Patients showed remarkable similarity in this ON dimension. However, there was a 72% probability that either a Dieter or Patient would score higher than a Student.

The EHQ Feelings subscale showed distinct patterns from Knowledge subscale in Patients and Dieters group. The Students group showed the lowest scores and almost the same slopes for each variable considered in the analysis.

Considering the positive component of perfectionism, higher values of EHQ-Feelings were highlighted partially overlaps between Patients and Dieters groups: when high levels of attempt to pursue perfection are shown, satisfaction belonging to strict adherence to diet is greater; people on diet who make efforts to reach the perfection, direct this goal within the scope of healthy eating and experience positive feelings when it is reached. This could represent a maintenance factor for ON.

When considering higher levels of the **Concern dimension of perfectionism (PC)**, an opposing trend was observed between the Dieters and Patients groups, with a significant interaction between the two. In Dieters, higher PC levels corresponded to an increase in ON-related emotions, while in Patients undergoing treatment for EDs, these levels were associated with a decrease in **EHQ-Feelings** scores. This suggests that for higher PC levels, Dieters display ON-related emotional patterns similar to Patients. Therefore, leading to maladaptive outcomes, both dimensions of perfectionism represent a risk factor for ON in people following a diet.

In the Dieters group, concerns about mistakes and high expectations were found to predict feelings of control over one’s diet and the efforts made to maintain it. Conversely, in the Patients group, higher levels of MPS-Concern were associated with lower ON-related emotions. Similar to the findings in the Knowledge dimension of ON, Dieters appeared to be egosyntonic with their feelings toward adhering to healthy eating habits, while individuals diagnosed with EDs were egodystonic due to the psychological and nutritional treatment they were undergoing. When comparing Dieters and Patients, a similar pattern emerged in the **Concern dimension of perfectionism**, as reflected in the total OCI-R scores. Both groups showed a partial overlap in ON-related emotions, with opposing relationships between obsessive-compulsive symptomatology and the perfectionistic subscales:

- Higher levels of obsessive-compulsive symptoms were associated with increased **EHQ-Feeling** scores in Dieters, suggesting that repetitive behaviors may serve as a coping strategy to maintain control over eating habits.
- In contrast, the same higher levels predicted lower **EHQ-Feeling** scores in Patients, likely indicative of psychological distress linked to maladaptive behaviors.

In the Dieters group, repetitive behaviors, rather than mere obsessiveness, may act as a behavioral solution to satisfy their need for control over eating habits. Both Dieters and Patients demonstrated a 93% probability of scoring higher on ON-related emotions than the Students group.

In conclusion, this is the first study in Orthorexia Nervosa that evaluates with Bayesian models the probability of ON related to perfectionism and obsessive-compulsive symptomatology in three groups. It was confirmed that the Patients group with a diagnosis of Eating Disorders have higher problems related to ON as compared to the Dieters and Students group. Moreover, the concern dimension of perfectionism affects all the groups at risk, while the striving dimension does not affect problems related to ON. Insights on knowledge and feelings dimensions of ON are necessary because they could represent important subsequent risk factors for their relationship with perfectionism and obsessive-compulsive features. In particular, in Dieters it is important to consider perfectionism and its possible impact on ON.

These results allow us to conclude that problematic ON is characteristic of patients with EDs, followed by patients on a diet and the student population at risk. Both positive and negative perfectionism have direct effects in all dimensions of ON, and obsessive-compulsive characteristics appear to be a constant construct related to ON.

There are some limitations of this study. Firstable, this is a cross-sectional study, which does not allow the evaluation of the evolution of the considered aspects over time. Longitudinal studies should be conducted. Moreover, the number of participants of each group is different from others, this is due to the difficulty of recruiting clinical population. Future studies should recruit more people on diet and more people suffering from EDs. The clinical sample is heterogeneous, this represents a limit regarding the investigation about the predictiveness of OCD symptoms, PS e PC in relation to ON in different EDs diagnosis. Future studies should consider the same hypothesis in different diagnoses of EDs (for example in AN, in BN, in BED).

## Electronic supplementary material

Below is the link to the electronic supplementary material.

Supplementary Material 1

## Data Availability

Data will be available upon reasonable request to the corresponding author who has full access to the data reported in the manuscript.

## Declarations

### Data access

Data will be available upon reasonable request to the corresponding author who has full access to the data reported in the manuscript.
