# Supplementary material for: What is more likely in orthorexia nervosa: perfectionism or OC symptoms? A bayesian method in clinical and non-clinical samples
Source: BMC Psychol. 2025 Mar 11;13:230. doi: 10.1186/s40359-025-02517-2 (PMC11899392; doi:10.1186/s40359-025-02517-2)

**List of Abbreviations**

**AN**: Anorexia Nervosa

**ANBP**: Anorexia Nervosa Binge-Eating/Purging subtype

B**ED**: Binge Eating Disorder

**BN**: Bulimia Nervosa

**ED**: Eating Disorder

**EDNOS**: Eating Disorder Not Otherwise Specified

**EHQ-21**: Eating Habits Questionnaire (21 items)

**LOO**: Leave-One-Out cross-validation criterion

**MCMC**: Markov Chain Monte Carlo

**MPS**: Multidimensional Perfectionism Scale

**OCI-R**: Obsessive Compulsive Inventory-Revised

**OCD**: Obsessive-Compulsive Disorder

**ON**: Orthorexia Nervosa

**OSFED**: Other Specified Feeding and Eating Disorders

**PC**: Perfectionism Concern / Perfectionistic Concerns

**PPC**: Posterior Predictive Check

**PPD**: Posterior Predictive Distribution

**PS**: Perfectionism Striving / Perfectionistic Strivings

**PSRF (Rhat)**: Potential Scale Reduction Statistic

**Supplementary Material**

The specific models that we compared are summarized in **Table 1.**

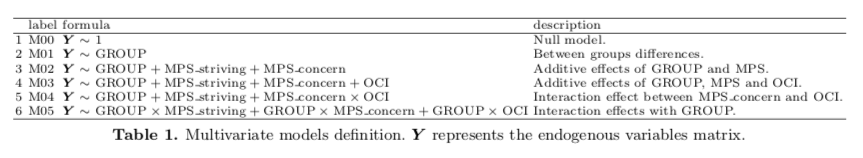


In **Table 2** are reported in detail all used priors in the six models. The class column indicates the type of parameter: b = regression coefficient, Intercept = model intercept, Lrescor = residuals correlations, sigma = residuals standard error. The coef column indicates the specific model coefficient name and the resp column reports the specific dependent (response) variable. The lb column reports the lower bound for parameter restriction; in particular in our case values zero indicate that priors for sigma parameters are truncated because these parameters can be only positive.


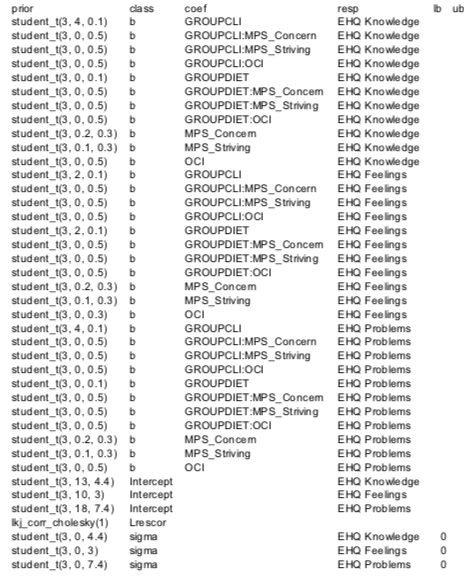

Supplement: Supplementary file 1 — Supplementary Material 1 [file 40359_2025_2517_MOESM1_ESM.docx]
